# Acute liver failure associated with Fructus Psoraleae: a case report and literature review

**DOI:** 10.1186/s12906-019-2493-9

**Published:** 2019-04-11

**Authors:** Ang Li, Minhong Gao, Na Zhao, Ping Li, Jinguo Zhu, Wenfei Li

**Affiliations:** 1 Department of Dermatology, Qianfoshan Hospital, Shandong University, 16766 Jing-Shi Road, Jinan, 250014 China; 2 Department of Orthopedic Surgery, The Sixth People’s Hospital Affiliated to Shanghai Jiao Tong University, Shanghai, China; 3 Department of Clinical Medicine, Queen Mary School, Medical school, Nanchang University, Nanchang, China; 4 Zhangqiu District People’s Hospital, Jinan, China; 5 Department of Clinical Medicine, Taishan Medical College, Tai’an, China; 6 Department of Dermatology, Laicheng District People’s Hospital, Laiwu, China

**Keywords:** Fructus Psoraleae, Qubaibabuqi tablets, Vitiligo, Hepatotoxicity

## Abstract

**Background:**

Fructus Psoraleae is the seed of *Psoralea corylifolia* Linn. Fructus Psoraleae has been shown to be effective in treating some skin diseases, such as vitiligo. As a main ingredient in five types of herbs in the Qubaibabuqi tablet formula, Fructus Psoraleae plays an important role in the treatment of vitiligo. Fructus Psoraleae has potential hepatotoxicity, thus Qubaibabuqi tablets also have potential liver toxicity.

**Case presentation:**

A 53-year-old woman who was diagnosed with vitiligo in September 2017 was treated with Qubaibabuqi tablets. After approximately 7 months of treatment, the patient developed a severe, diffuse yellow staining of the skin and sclera in March 2018. On admission, she was diagnosed with acute cholestatic hepatitis associated with Fructus Psoraleae. Despite receiving active treatment, her condition rapidly deteriorated and she died 5 days later due to acute liver failure and multiple organ dysfunction. To the best of our knowledge, there are only six reported cases of liver injury associated with Fructus Psoraleae described in the English language literature; however, cases of acute liver failure associated with the use of Fructus Psoraleae have not been described.

**Conclusion:**

As a main ingredient in the Qubaibabuqi tablet formula, Fructus Psoraleae has potential hepatotoxicity. This potentially fatal adverse effect should be considered when physicians prescribe Qubaibabuqi tablets.

## Background

Fructus Psoraleae, is the seed of *Psoralea corylifolia* Linn, and is a constituent of many herbal formulas. Qubaibabuqi tablets are a traditional Uyghur herbal medicine, and its formula includes five types of herbs (Fructus Psoraleae, *Vernonia anthelmintica*, *Alpinia officinarum*, *Operculina turpethum*, and *Plumbago zeylanica*) [1]. Fructus Psoraleae, is the main ingredient of Qubaibabuqi tablets and has long been effective in the treatment of vitiligo [2]. Fructus Psoraleae has the following chemical constituents: psoralen, isopsoralen, 8-methoxypsoralen, bakuchicin, coumestrol, psoralidin, corylidin, and agtragalin. Thus, Fructus Psoraleae plays an important role in the treatment of vitiligo with Qubaibabuqi tablets. Some reports have shown that Fructus Psoraleae is a potential hepatotoxin [1]. There are no reports of liver toxicity associated with the other four herbs in the Qubaibabuqi tablet *formula. Therefore, we consider that* the hepatotoxicity associated with Qubaibabuqi tablets are due to Fructus Psoraleae. *Acute liver* failure can be *fatal* and is characterized by a sudden loss of hepatic function [3]. The main features of acute liver failure are rapid-onset jaundice, weakness, and other symptoms. These symptoms indicate that the liver has sustained severe damage in a previously healthy individual.

Herein we describe a case of *acute liver* failure in a woman diagnosed with vitiligo following treatment with Qubaibabuqi tablets. Several cases of liver injury associated with the use of Qubaibabuqi tablets or Fructus Psoraleae have been reported [1, 4–6]; however, cases of acute liver failure associated with the use of Qubaibabuqi tablets or Fructus Psoraleae have not been described.

## Case presentation

A 53-year-old woman was diagnosed with vitiligo in September 2017 and was treated with oral Qubaibabuqi tablets (15 tablets three times daily; Xinjiang Yinduolan Uyghur Pharmaceutical Company Limited, Urumqi, China), 10 mg of prednisone acetate tablets (Xinhua Pharmaceutical Company Limited, Zibo, China) once daily, and narrowband-ultraviolet B (NB-UVB) phototherapy (Sigma household narrowband-ultraviolet phototherapy instrument [SS-01B] pocket portable; Shanghai *Sigma* High Technology Co., Ltd. Shanghai, China) every other day. The prednisone acetate tablets were self-discontinued 3 months later; however, she continued to take Qubaibabuqi tablets orally and NB-UVB phototherapy was undertaken at home. The patient seldom saw her physician, and bought Qubaibabuqi tablets from a pharmacy over the next 7 months. The patient presented to our clinic on March 27, 2018. She complained of weakness, nausea, and vomiting for 3 days. She was admitted to the Gastroenterology Department.

Physical examination revealed severe, diffuse yellow staining of the skin and sclera, and bilateral lower extremity edema. Patches of vitiligo involved the left frontal region, left chest, and right lower abdomen. The skin lesion in the left frontal region was nearly normal, and the color of the surrounding skin lesions on the left chest and right lower abdomen were black due to therapy. She had no history of cigarette smoking, alcohol consumption, or autoimmune *diseases*. She was otherwise healthy and denied taking any medications other than Qubaibabuqi tablets. A liver biopsy after hospitalization showed acute cholestatic hepatitis. Additional laboratory analyses revealed the following: normal routine blood tests; alanine aminotransferase (804.40 U/L; normal 7–40 U/L); aspartate aminotransferase (896.30 U/L; normal 13–35 U/L); total bilirubin (335.20 μmol/L; normal 5.00–24.00 μmol/L); direct bilirubin (233.00 μmol/L; normal 0.24–7.10 μmol/L); and indirect bilirubin (102.20 μmol/L; normal 2.80–23.80 μmol/L), negative HIV and syphilis antibodies; normal hepatitis A, B, and C serology and negative Epstein-Barr virus, herpes simplex virus, and human cytomegalovirus. Causality assessment using the updated Council for International Organizations of Medical Sciences scale showed 10 points (a probable causality) for Fructus Psoraleae contained in Qubaibabuqi tablets. Based on these data, the patient was diagnosed with drug-induced hepatitis.

After admission, the patient was given a high carbohydrate, low fat, and moderate protein diet, and actively maintained a balance of water, electrolytes, and pH, and corrected hypoproteinemia. Measures to prevent infection and provide oral care were adopted. She actively used medications (Magnesium Isoglycyrrhizinate Injection, Ornithine Aspartate Injection and Ademetionine 1, 4-Butanedisulfonate for Injection, and so on.) to protect liver function and treat hepatic coma. Due to poor treatment efficacy, the patient’s condition was aggravated and she was treated with an artificial liver support system besides the routine medicinal therapy. Although the patient was given active treatment, her condition continued to deteriorate. Her spirit gradually changed from sobriety to wandering, and then coma. Some symptoms indicated that her life was in danger, and these symptoms included fetor hepaticus, flapping tremor, multiple patches of flaky ecchymoses, elevated liver enzymes, and a coagulopathy. She died 5 days later due to acute liver failure and multiple organ dysfunction. This serious adverse event has been reported to the China Food and Drug Administration through the National *Adverse Drug Reaction Surveillance System (No. 3701011011107321800095).*

## Discussion and conclusions

*Psoralea corylifolia* Linn (also known as Bu-gu-zh, Bu Ku Zi, Bol-gol-zhee, Boh-Gol-Zhee, Babchi, and Bakuchi) is a plant grown in China, India, Sri Lanka, Burma, and other countries, which is considered an important herbal medicine. Some herbal formulas have definite therapeutic effects in the treatment of skin diseases [7, 8]. Many Chinese herbal formulas have been successfully used to treat vitiligo, such as the Uygur medicine Qubaibabuqi tablets. However, there are some reports of adverse reactions and hepatotoxicity of Qubaibabuqi in China, and one study showed liver, kidney, and reproductive toxicity of Fructus Psoraleae [9].

Liver injury caused by Fructus Psoraleae was originally described by Nam et al. in 2005, and since then there have only been six cases of liver injury associated with Fructus Psoraleae described in the English language literature [1, 4–6]. The clinical features seen in these patients are listed in Table 1. Our patient presented with severe, diffuse yellow staining of the skin and sclera, and progressive deterioration in liver function following treatment with oral Qubaibabuqi tablets. The patient died 5 days after admission due to acute liver failure and multiple organ dysfunction. Compared with the six cases previously reported, our patient is the first case to die, likely due to Fructus Psoraleae-induced liver toxicity.

**Table 1:** Comparison of cases of liver injury caused by Psoraleae Fructusinthe literature

Case	Author (year)	Age (year)/sex	Chief features	Dosage	Duration	Prognosis
1	Nam SW et al. 2005 [6]	44/F	Nausea, vomiting, and general weakness for 1 month	A large dose	One week	Good
2	Cheung WI et al. 2009 [7]	39/M	Jaundice, anorexia, and abdominal discomfort for 1 week	Normal	Two months	Better
3	Cheung WI et al. 2009 [7]	22/M	Jaundice and tea-colored urine for 3 days	Normal	Two months	Good
4	Cheung WI et al. 2009 [7]	20/F	Fever, epigastric pain, and jaundice for 1 week, and palpable liver edge on examination	Normal	Two weeks	Better
5	Teschke R et al. 2009 [3]	64/F	Jaundice in the sclera	Normal	Nine months	Good
6	Smith DA et al. 2014	52/F	Jaundice, vomiting, pruritus and abdominal pain for 1 week.	/	/	Good

“/” indicates no description in the article

The mechanism underlying Fructus Psoraleae-associated liver damage is complex. One study indicated that psoralen induced inhibition of the mTOR signaling pathway and mitochondrial injury, then hepatotoxicity resulted [10]. The vitamin D-vitamin D nuclear receptor axis plays a protective role in 8-MOP-induced hepatotoxicity [11], and another study showed that 8-MOP disturbs phospholipid efflux and bile acid homeostasis and induces cholestatic liver injury [12]. One investigation showed that humanuridine 5′-diphospho-glucuronosyltransferase 1A1 (UGT1A1) is responsible for metabolic elimination of bilirubin, and Fructus Psoraleae extract and its major constituents have inhibitory effects on UGT1A1. This is an important reason for triggering Fructus Psoraleae-associated hepatotoxicity, including elevated bilirubin levels and liver injury [13]. It was previously reported that Fructus Psoraleae extract, such as psoralens and bakukiol, induce cholestatic hepatotoxicity, suggesting potential hepatotoxicity [14]. Another study suggested that liver injury associated with Fructus Psoraleae may be related to large doses of Fructus Psoraleae in rats [15]. There are no reports of liver damage caused by NB-UVB. Thus, our patient who had severe hepatotoxicity and died of acute liver failure and multiple organ dysfunction, this may have been due to large doses and the toxic accumulation of Fructus Psoraleae as she had been taking 15 tablets of Qubaibabuqi three times daily for approximately 7 months.

Vitiligo is a common skin disease and is not life-threatening. The patient died of acute liver failure and multiple organ dysfunction, likely due to Qubaibabuqi tablet treatment for vitiligo. Some lessons can be learned from this case. First, physicians should be cautious when they prescribe medicines with potential hepatotoxicity. *Furthermore,* physicians should improve patient medical health and safety education in those who require long-term treatment. Second, patients should be instructed to see their physician and undergo regular examinations. Third, the pharmacist should fulfill their professional responsibilities. If these points are followed we can prevent similar incidents occurring.

This is the first case of a patient dying, likely due to Fructus Psoraleae a component of Qubaibabuqi tablets. Further studies are needed to define the exact mechanism of Fructus Psoraleae hepatotoxicity. The cautious use of herbal products with potential hepatotoxicity, such as Qubaibabuqi tablets or other herbal medicines which contain Fructus Psoraleae, is essential.

## Abbreviations

NB-UVB: narrowband-ultraviolet B
UGT1A1: uridine 5′-diphospho-glucuronosyltransferase 1A1
